# Building crypto portfolios with agentic AI

**DOI:** 10.3389/frai.2026.1871825

**Published:** 2026-10-08

**Authors:** Paolo Giudici, Alessandro Piergallini, Antonino Castelli

**Affiliations:** Department of Economics and Management, University of Pavia, Pavia, Italy

**Keywords:** crypto assets, machine learning, multi-agent system, portfolio optimization, trustworthy AI

## Abstract

The rapid growth of crypto markets is increasing investment opportunities, but also volatility risks. There is a need to automate portfolio construction to rapidly adapt investment decisions to rapidly changing markets. To fill the gap, in this paper we propose a multi-agent system designed to autonomously construct and evaluate crypto-asset allocations, in terms of returns and risks. We exemplify the methodology by its application to daily frequency return data of the ten most capitalized cryptocurrencies, from 2020 to 2025. Specifically, we compare two automated investment strategies: a static strategy and a rolling-window optimization strategy, both based on the optimisation of evaluation metrics such as the Expected Return and Sharpe ratio. The results show that the dynamic optimization strategy achieves better performance in terms of risk-adjusted returns, both in-sample and out-of-sample. This highlights the benefits of multi-agent systems in portfolio management, particularly in volatile markets such as cryptocurrency markets.

## 1 Introduction

Cryptocurrencies have recently grown from a niche phenomenon to a mainstream asset class. While they promise high returns, their high volatility, and frequent regime shifts calls for the construction of automated portfolios that can rapidly adapt to a changing environment.

To this aim, recent progress in Multi-Agent Systems (MAS) offers a modular way to automate investment decisions in crypto asset markets. Automated agents can rapidly ingest, clean and analyze data, construct portfolios and report results collaboratively. MAS have been recently shown to outperform tradtional portfolios in real trading environments, see Xiao et al. (2025).

MAS rely on Large Language Models (LLM), merging qualitative judgment with quantitative signals for asset pricing, as in the AAPM framework proposed in Cheng and Chin (2024). Recent works have shown that LLM models can help to improve transparency and explainability of financial decisions Hadji Misheva et al. (2021). LLM agents have been employed to simulate human-like economic behavior for scalable virtual experiments Horton (2023), and to improve anomaly detection and real-time risk management in financial markets Park (2024). However, despite the growing literature that underlines the importance of LLM for explainable portfolio decision making, there is not yet evidence on the importance of MAS based on LLM and, in particular, for crypto-asset portfolio management.

This paper fills the gap, proposing a MAS model, implemented with the CrewAI software, to build a portfolio of crypto assets. Such a model can indeed deliver effective, scalable and auditable portfolios, and produce workflows more transparent, auditable, and accessible, and not only to a small group of specialists. The novelty of our contribution is not the definition of a new multi-agent learning model. Rather, it lies in the automated construction and execution of an end-to-end MAS for portfolio management using LLM-based agents. More specifically, following recent advances in multi-agent architectures Masterman et al. (2024); Zhang and Liu (2024); Fu et al. (2024); Zhang (2025), we will compare two MAS-based crypto-allocation strategies, showing that dynamic rebalancing improves performance with respect to a static strategy. The rest of the paper is organized as follows. The next Section presents the data that motivates our work. Section 3 specifies our proposed MAS model, and Section 4 how it can be employed to build two portfolio allocation strategies. Section 5 describes the results of the application of the strategies to the data, and Section 6 concludes.

## 2 Literature review

In financial applications, LLMs can be utilized for economical analysis because they can give responses similar to what we might expect from a human by processing a wide range of textual inputs. From this viewpoint, the paper Horton (2023) examines how LLMs, like GPT-3, can simulate human like economic behavior, allowing researchers to conduct virtual economic experiments. These simulations mirror traditional economic studies, offering a scalable, cost-effective tool for testing hypotheses with real world experiments, though with some limitations regarding data quality and representativeness.

A related paper is Li et al. (2023), which introduces a new approach to macroeconomic simulation using LLM-powered multi-agent systems. EconAgent creates agents that can simulate human-like economic behaviors, including decisions in the labor, consumption, and financial markets. The framework addresses key challenges in macroeconomic simulations, such as agent heterogeneity and the influence of macroeconomic trends. The method also offers adaptability and realistic decision-making, exhibiting improved simulations over traditional models, potentially transforming macroeconomic policy analysis and research.

Multi-agent systems can be used also for anomaly detection in financial services, as shown in Park (2024), which presents an LLM-based multi-agent framework for anomaly detection in finance. Agents are defined for specific tasks, such as data validation, external information gathering, and institutional knowledge integration. These agents collaborate to identify, analyze, and validate financial data anomalies efficiently. This framework enhances accuracy and reduces human involvement, making it useful for real-time monitoring and decision-making in financial markets, where quick and reliable anomaly detection is crucial.

Understanding and monitoring multi-agent LLM systems is crucial because their interactions can amplify biases, leading to unintended behaviors and causing potential harm; hence, it is essential to ensure that agents are built to be fair, safe, and ethical by design in their application to complex tasks.

From this perspective, Zhou et al. (2020) explored the coordination of lender and borrower agents to optimize credit risk assessment. Chen et al. (2024) have proposed decentralized trust mechanisms among agents to improve transparency and mitigate fraud.

However, despite these advancements, several critical challenges remain unaddressed: integration of heterogeneous data sources, interpretability of agent decisions, and regulatory compliance for multi-agent environments.

In particular, few works have systematically examined how multi-agent architectures can be holistically integrated into the end-to-end financial pipeline—from application intake to risk pricing and financial monitoring, ensuring trustworthy decisions. Moreover, the interplay between cooperative and competitive agent behaviors in such contexts is still an underexplored area, especially in relation to fairness, accountability, and robustness.

The safety of multi-agent systems is important, especially when it involves the financial services industry, which is heavily regulated. As Chan et al. (2023) describe, harms in agentic systems may lead to systemic and long-range impacts, undermining collective decision-making power. Indeed, the research on FATE (Fairness, Accountability, Transparency, and Ethics) Babaei et al. (2025), suggests that as programmable systems become more agentic, they may amplify biases and inequities, particularly for marginalized groups. Some other challenges, not necessarily related to harm and safety, were elucidated by Han et al. (2024). These include, but are not limited to, the following: optimizing task planning,managing complex context information, and improving memory management. Identifying the challenges of LLM-powered multi-agent systems and their potential solutions is of paramount importance in ensuring the safety and compliance of agentic systems. Some of these challenges include error handling techniques and/or system failures leading to unpredictable behavior like hallucination, lack of control and oversight over the input variables that shape the decision of the system, induced preference or bias, toxic degeneration, and difficulty assessing how agents reach their conclusions.

## 3 Data

The dataset we consider to illustrate our methodology consists of the daily closing prices of the ten most capitalized cryptocurrencies, for the time period that goes from 2020-08-20 to 2025-03-13. The selected cryptocurrencies are: Bitcoin (BTC), Ethereum (ETH), Binance Coin (BNB), Solana (SOL), Ripple (XRP), Cardano (ADA), Dogecoin (DOGE), Avalanche (AVAX), Polkadot (DOT), and Shiba Inu (SHIB). The time period has been chosen going backward, from the day in which we recorded the most capitalized crypto assets (2025-03-13) back to when all such cryptos existed (2020-08-20).

The descriptive statistics that summarize the prices of the selected cryptocurrencies, for the considered period, are reported in Table 1.

**Table 1:** Descriptive statistics of selected cryptocurrencies (USD).

Asset	Mean	Median	Standard deviation	Min	Max
BTC	41770.30	21685.75	21000.27	15787.28	106146.27
ETH	2316.82	855.93	4812.09	993.64	2443.43
BNB	335.07	152.84	750.27	40.99	396.41
SOL	56.34	69.76	261.87	3.69	87.21
XRP	0.56	0.55	3.30	0.25	0.76
DOGE	0.10	0.10	0.68	0.01	0.14
ADA	0.50	0.56	2.97	0.24	0.77
AVAX	25.72	24.40	134.53	8.79	33.52
SHIB	0.01	0.01	0.00008	0.01	0.03
DOT	7.02	11.27	53.88	3.65	12.81

Table 1 highlights the challenge of the analysis: while Bitcoin's mean price exceeds USD 40,000 with a standard deviation above USD 21,000, mid-cap tokens such as SHIB have a much lower variability.

Table 2 summarizes the risk characteristics of the daily returns from the 10 cryptocurrencies.

**Table 2:** Annualized volatility and historical VaR of cryptocurrency log-returns.

Asset	Annualized volatility	VaR (95%)	VaR (99%)
BTC	0.5083	0.0512	0.0910
ETH	0.6661	0.0666	0.1230
BNB	0.7149	0.0615	0.1159
SOL	1.0337	0.0901	0.1692
XRP	0.9036	0.0745	0.1377
DOGE	1.1958	0.0828	0.1648
ADA	0.8323	0.0767	0.1279
AVAX	1.0014	0.0954	0.1588
SHIB	1.5595	0.1178	0.1823
DOT	0.8771	0.0819	0.1372

Table 2 shows that Bitcoin records the lowest annualized volatility (50.8%) and the smallest Values at Risk (VaR), while several alterative coins, notably SHIB, DOGE, SOL and AVAX, exhibit substantially higher dispersion and historical VaR levels that may exceed 12–18%. These results confirm the heavy-tailed and highly heterogeneous nature of cryptocurrency returns, with significant implications for risk management and portfolio allocation.

We now further investigate the behavior of the Bitcoin, the dominant asset in the crypto universe, and the benchmark for crypto market behavior, in Figure 1.

**Figure 1:**
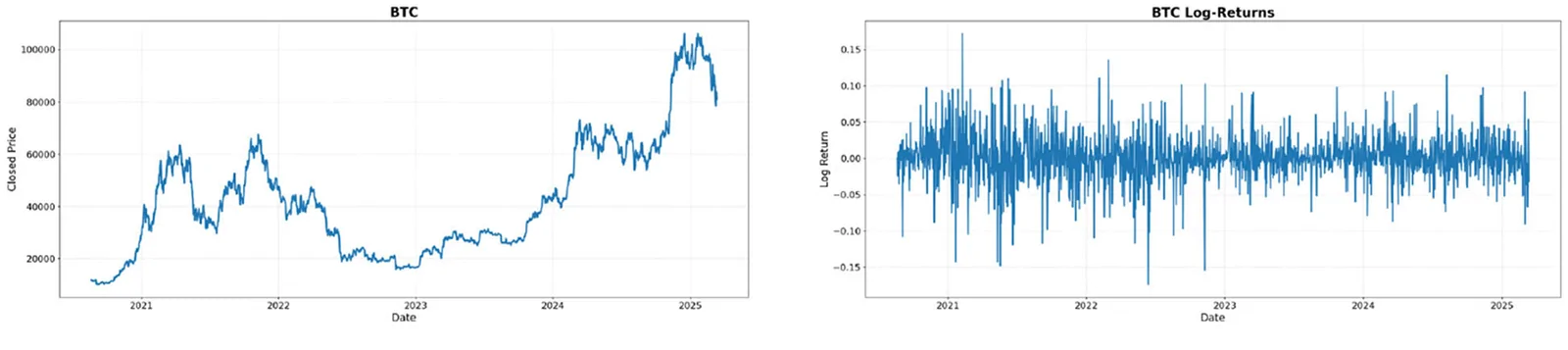
Bitcoin prices and returns comparison (2020–2025). **(a)** Bitcoin close prices from 2020 to 2025. **(b)** BTC returns from 2020 to 2025.

Figure 1 shows that Bitcoin price exhibits a strong boom-bust cycle in the period 2020–2025, with peaks in November 2021, followed by a marked correction. The returns of BTC display clear volatility clustering, alternating periods of high and low variance. This supports the need for a dynamic portfolio allocation framework, as the large fluctuations in asset-specific volatility and return behavior over time challenge the assumptions of constant risk and correlation used in static models.

We now consider the correlations among the considered crypto assets, in Table 3 .

**Table 3:** Correlation matrix of daily log-returns.

Cryptoasset	BTC	ETH	BNB	SOL	XRP	DOGE	ADA	AVAX	SHIB	DOT
BTC	1.000	0.811	0.649	0.562	0.553	0.509	0.659	0.604	0.389	0.662
ETH	0.811	1.000	0.675	0.636	0.583	0.480	0.703	0.638	0.388	0.723
BNB	0.649	0.675	1.000	0.560	0.504	0.357	0.577	0.587	0.338	0.611
SOL	0.562	0.636	0.560	1.000	0.479	0.359	0.568	0.609	0.325	0.579
XRP	0.553	0.583	0.504	0.479	1.000	0.371	0.611	0.522	0.287	0.542
DOGE	0.509	0.480	0.357	0.359	0.371	1.000	0.454	0.413	0.392	0.433
ADA	0.659	0.703	0.577	0.568	0.611	0.454	1.000	0.670	0.316	0.689
AVAX	0.604	0.638	0.587	0.609	0.522	0.413	0.670	1.000	0.308	0.665
SHIB	0.389	0.388	0.338	0.325	0.287	0.392	0.316	0.308	1.000	0.357
DOT	0.662	0.723	0.611	0.579	0.542	0.433	0.689	0.665	0.357	1.000

Table 3 confirms the strong interconnectedness of the cryptocurrency market. Bitcoin and Ethereum exhibit the strongest bilateral correlation (0.81), confirming their central role within the digital asset ecosystem. Major altcoins such as ADA, DOT, BNB, SOL and AVAX also display substantial positive correlations, generally ranging between 0.52 and 0.78, suggesting that diversification opportunities across large-cap cryptocurrencies remain limited.

SHIB exhibits comparatively lower correlations, ranging between 0.29 and 0.39 with the remaining assets. While this indicates a stronger idiosyncratic component relative to the rest of the market, SHIB still remains positively associated with overall cryptocurrency dynamics. Overall, the correlation structure highlights the presence of dominant market factors affecting most digital assets, reinforcing the importance of dynamic portfolio allocation and risk management techniques.

## 4 Multi agent systems

The portfolio model we propose is based on a Multi-Agent System (MAS) framework that uses Crew AI, a software platform which permits agents to interact in a modular way, across distinct stages of the investment pipeline. Each agent is programmed with a specific objective, works with a pre-specified tool to reach its objective, and communicates with others through structured prompts. The model is defined by the following eight tools.

- data_loader_tool, which nretrieves and formats the crypto dataset;
- data_cleaner_tool, which handles missing values and anomalies;
- data_splitter_tool, which splits the data into training and testing subsets, using a 80%/20% rule;
- portfolio_metrics_tool, which computes key performance metrics such as Annualized Return, Volatility, Sharpe and Sortino ratios;
- portfolio_optimizer_tool, which performs static portfolio optimization using the mean-variance criteria;
- rolling_portfolio_optimizer_tool, which performs a dynamic portfolio optimization using a 30-day rolling window Sharpe ratio maximization with no short-selling;
- portfolio_metrics_with_benchmark_tool, compares crew performance with benchmark, S&P500;
- file_checker_tool, validates logical coherence of agents outputs.

Each tool is mapped to one agent, and the agents collaborate with each other, according to the flow defined in Figure 2:

**Figure 2:**
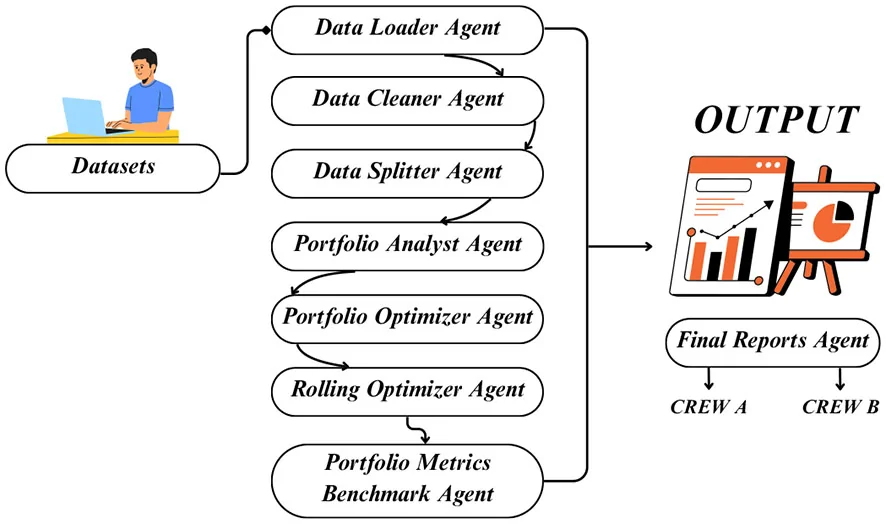
Rrepresentation of the multi-agent system model.

As shown in Figure 2, the agents are organized into two operational crews, with the same structure but a different optimization logic. Crew A performs a static portfolio analysis: it starts with equal weights and applies a single mean-variance optimization on the training set. Crew B adopts a rolling optimization, recalculating weights every 30 days, to reflect the evolving market conditions.

Each crew communicates the chosen portfolio by means of a Final Report Agent, which does not use any tool since its role is mainly interpretative. It integrates outputs from all previous agents into a single performance evaluation.

The proposed MAS model design guarantees modularity, replicability, and transparency, making the process not only actionable, but also fully auditable by supervisors.

### 4.1 Agentic architecture and role of LLMs

The proposed framework is agentic in an operational sense: once the workflow is launched, the CrewAI agents execute the assigned tasks autonomously, invoke their dedicated tools, exchange intermediate outputs, and produce the final portfolio reports without further human intervention. The human designer defines the agents, tasks, tools, and execution order ex ante, while the agents autonomously perform the data loading, cleaning, splitting, optimization, evaluation, and reporting stages.

The role of LLMs is not to directly estimate portfolio weights or replace the numerical optimizer. Portfolio weights are computed deterministically by the optimization tools. Instead, LLM-based agents orchestrate task execution, interpret intermediate outputs, transfer information between tasks, validate consistency, and generate human-readable reports. The proposed framework therefore combines deterministic quantitative tools with autonomous LLM-based workflow execution and communication, improving transparency, auditability, and reproducibility.

Indeed, the workflow of the proposed model is totally transparent: all parameters, constraints, and intermediate computations are explicit, and ensure reproducibility. No randomness is introduced during flow execution.

For the sake of clarity, we now report a more precise description of the steps of the MAS model in Figure 2.

Before the process begins, an agent responsible for checking the presence and accessibility of the required input files verifies the availability of the main dataset. This ensures that the pipeline can proceed safely, before any data manipulation begins.

1. **Dataset loading:** a dedicated data importing agent is tasked with reading the dataset, supporting both CSV and Excel formats. After loading, the agent reports the number of rows and columns and stores the results locally. This path becomes the shared reference for subsequent agents in the workflow.
2. **Dataset cleaning:** a data-cleaning agent replaces missing values (NaNs) using the previous day's observation. The cleaned dataset replaces the original, ensuring downstream consistency without the need to track separate versions.
3. **Train-test splitting:** a splitting agent divides the cleaned dataset into two parts: 80% for training and 20% for testing. This ratio is controlled via a fixed parameter set to 0.8. The split is deterministic and preserves the structure and column ordering of the original data.
4. **Evaluation of equal-weight strategy on training set:** a portfolio analysis agent evaluates the training dataset assuming equal weights for all cryptoassets. This evaluation computes the following risk and performance indicators:

- Annualized expected return.
- Annualized volatility.
- Sharpe ratio, assuming a risk-free rate of 1%.
- Sortino ratio.
- Maximum drawdown.
- Alpha (excess return).
- Liquidity risk, measured via cross-sectional volatility dispersion across assets.
- Regime change detection, based on volatility shifts greater than a threshold of 0.15.

Liquidity risk is proxied by the dispersion of asset-specific return volatilities: higher heterogeneity among volatilities indicates lower market depth and, therefore, higher portfolio liquidity risk. The agent saves a report with all computed metrics in Markdown format.
5. **Portfolio optimization:** an optimization agent computes the optimal asset allocation by maximizing the Sharpe ratio, under the following constraints:

- Full investment constraint: weights must sum to 1.
- Long-only constraint: weights must lie in the range [0, 1].
- Optimization method: Sequential Least Squares Programming (SLSQP).

The optimized portfolio is evaluated in terms of expected returns, volatility, and both Sharpe and Sortino ratios. The resulting weights are saved for later reuse.
6. **Evaluation of optimized portfolio on training set:** using the optimized weights, another agent build the portfolio and evaluates portfolio performance on the training set, this time comparing the portfolio against a benchmark series. In addition to the metrics already computed for the equal-weight strategy, this step also includes:

- Alpha and beta, estimated via linear regression on benchmark returns.
- Net expected return, adjusted for transaction costs (0.1% per trade, assuming a 5% daily turnover).

The output is a second Markdown report detailing the metrics of the optimized strategy on the training data.
7. **Generalization test on the test set:** the same agent is used again to evaluate how the optimized portfolio performs on the test dataset. It uses the previously computed weights and the same benchmark reference to estimate generalization capability. A third Markdown report is produced.
8. **Final strategy comparison and recommendation:** a final report agent integrates the three reports described above: one for the equal-weight strategy on the training set, one for the optimized strategy on the training set, and one for the optimized strategy on the test set. This agent compares the two approaches in terms of return, risk, and drawdown metrics, then evaluates whether the optimized strategy generalizes well to unseen data. It concludes with a recommendation on whether the optimized portfolio should be adopted going forward.

We finally remark that each agent in the pipeline operates deterministically, with no use of sampling or random initializations.

## 5 Portfolio construction

We now describe the practical implementation of the proposed model. This requires to specify the two alternative portfolio optimisation strategies followed, respectively, by Crew A and Crew B. To this end, in subsection 4.1, we discuss a static model (Crew A), while in subsection 4.2 we discuss a dynamic model, with rolling optimization (Crew B). Both approaches rely on the same dataset, agent framework, and risk-adjusted performance evaluation pipeline, but differ in their timing, rebalancing logic and adaptivity, defined by the optimizer agent. In summary, in Crew A the optimizer behaves as a time static optimizer while in Crew B as a time dynamic optimizer.

### 5.1 Crew A: static portfolios

Crew A is designed to serve as a benchmark, as it applies a traditional investment strategy. Specifically, it first evaluates the performance of an equally weighted (1/*N*) portfolio across all 10 cryptocurrencies in the training set. Second, it applies a mean-variance optimization to determine the portfolio weights that maximize the Sharpe ratio, while minimizing the volatility, under the constraint of no short-selling and full investment. The sum of the weights will be equal to 1, meaning that each asset will have a weight value between 0 and 1. This strategy does not allow for portfolio rebalancing during the investment horizon.

The optimization based on the Sharpe ratio is performed once, using the entire training dataset, and the resulting portfolio is maintained constant throughout in-sample and out-of-sample periods. We recall that the *Sharpe Ratio* is computed as:


$$\begin{array}{c} \text{Sharpe}=\frac{E\left[R_{M}\right]-R_{f}}{\sigma_{M}} \end{array}$$ (1)

where *E*[*R*_*M*_] is the annualized expected return of the market, and σ_*M*_ represents its annualized standard deviation. All values are derived from daily data and rescaled by the number of trading days in a year, $\sqrt{252}$. The *volatility* is then calculated as:


$$\begin{array}{c} \sigma_{M}=\text{std}\left(R_{M}\right)\times \sqrt{252} \end{array}$$ (2)

### 5.2 Crew B: dynamic portfolios

Crew B extends the static approach of Crew A by incorporating a dynamic rebalancing. Specifically, it performs a rolling-window optimization in which every 30 days the most recent 30-day window of historical data is used to recompute the optimal portfolio weights. Figure 3 shows how the rolling window optimisation of Crew B works, for the considered time period.

**Figure 3:**
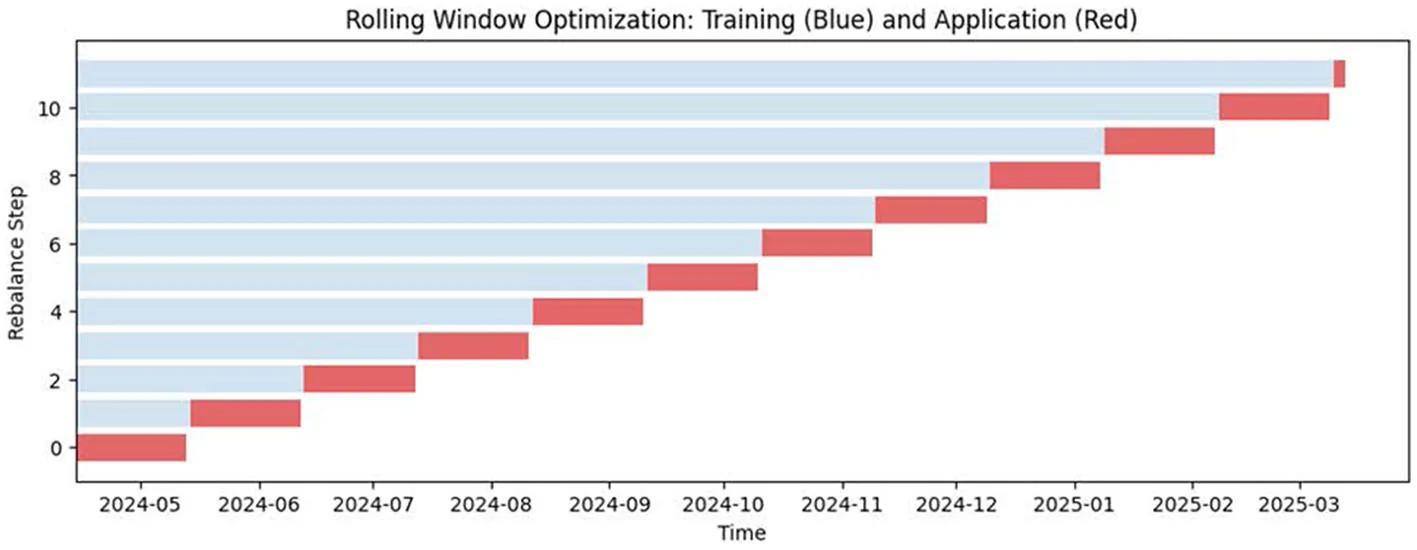
Description of Crew B optimization.

Figure 3 shows, for any given rebalancing date, the temporal window employed for the estimation of the portfolio weights (training period) and the subsequent interval in which such weights are applied out of sample (test period).

From Figure 3 it follows that the optimized portfolio is recalculated on a 30-day rolling basis, maximizing the Sharpe ratio subject to full-investment and no-short selling constraints. Indeed, rolling window techniques, widely adopted in portfolio allocation problems, have demonstrated significant benefits in capturing the time-varying dynamics of asset returns, see e.g. Chen (2024).

Note that the dynamic optimization of Crew B leads to a time series of portfolio weights, each adapted to the most recent market conditions. The updated weights are applied for the next 30-day period, after which the process repeats with newly updated data. This strategy reflects the reality of adaptive asset management, where investors revise allocations based on observed changes in returns, volatility, and correlations. In such a dynamic setting, the use of multi-agent reinforcement learning frameworks in portfolio optimization enables decentralized, adaptive, and goal-aligned trading policies, as explored in recent works on multi agent systems Lee et al. (2020a); Lin et al. (2022); Lee et al. (2020b); Cheng and Sun (2024)).

The rolling framework is particularly suited to the crypto environment, where regime shifts and sudden volatility spikes are frequent. Compared to Crew A, the rolling optimization captures temporal heterogeneity in risk-return profiles, potentially enhancing out-of-sample robustness and risk control.

Against these advantages, there is the disadvantage of higher transaction costs, which may however be offset by higher returns.

Table 4 summarizes and compares the asset weights obtained under a static optimization (Crew A), and the average asset weights obtained under a dynamic rolling optimization (Crew B). For the sake of completeness, we also report the equal weights setting.

**Table 4:** Comparison of asset weights for Crew A and Crew B.

Asset	Equal weights	Crew A	Crew B
BTC	0.1000	0.0000	0.0955
ETH	0.1000	0.0000	0.0881
BNB	0.1000	0.0000	0.1213
SOL	0.1000	0.6490	0.1271
XRP	0.1000	0.0000	0.0772
DOGE	0.1000	0.0411	0.0775
ADA	0.1000	0.0000	0.0663
AVAX	0.1000	0.0000	0.1848
SHIB	0.1000	0.3099	0.1375
DOT	0.1000	0.0000	0.0248

Table 4 underlines that, as expected from the nature of the data, Crew A weights are much more concentrated than the average Crew B weights.

#### 5.2.1 Sensitivity analysis

To assess the robustness of the rolling-window optimization framework and to provide an economic justification for the choice of the rebalancing horizon, we conduct a sensitivity analysis across alternative window lengths, respectively of 7, 14, 30, 60, and 90 days, and report it in Table 5.

**Table 5:** Sensitivity analysis of rolling-window lengths.

Window (days)	Exp. Return (%)	Volatility (%)	Sharpe	Sortino	Max drawdown (%)	Avg. turnover
7	-21.41	39.60	-0.54	-0.87	-48.31	0.048
14	-21.62	39.60	-0.55	-0.88	-48.29	0.067
30	-20.58	39.88	-0.52	-0.83	-48.69	0.098
60	-22.34	39.79	-0.56	-0.90	-49.04	0.126
90	-21.11	39.48	-0.53	-0.87	-47.51	0.139

Table 5 reports the out-of-sample performance metrics for each window length, including expected return, volatility, Sharpe ratio, Sortino ratio, and maximum drawdown. Across all specifications, results are qualitatively stable: risk-adjusted performance remains consistently weak, and no particular window length dominates in terms of return or downside risk. This evidence indicates that the empirical findings are not driven by a specific choice of rebalancing frequency. This mitigates concerns related to parameter tuning or ex post selection of the rolling horizon.

From an economic perspective, the similarity in performance across different window lengths reflects a trade-off inherent in rolling optimization strategies. Shorter windows react more rapidly to recent market conditions, but rely on noisier estimates, while longer windows provide smoother parameter estimates, at the cost of slower adaptation to regime changes. In particular, in high-volatility regimes shorter windows are preferable to improve responsiveness, whereas in low-volatility regimes longer windows may be preferable to reduce estimation noise. The intermediate horizons (30–60 days) offer a balanced compromise between responsiveness and estimation stability, consistent with common practices in dynamic portfolio management.

To further evaluate the practical implementability of the rolling strategy, we additionally report the average portfolio turnover associated with each window length. Formally, the portfolio turnover at a rebalancing date *t* is computed as:


$$\begin{array}{c} \text{Turnove}{\text{r}}_{t}=\overset{N}{\underset{i=1}{\sum }}|w_{i,t}-w_{i,t-1}|, \end{array}$$ (3)

where *w*_*i, t*_ denotes the portfolio weight of asset *i* at time *t*. In Table 5 we report the average turnover across all rebalancing dates for each rolling window length.

Table 5 shows that, as expected, given the considered period, all expected returns are heavily negative. Longer windows are associated with higher average turnover, reflecting larger portfolio reallocations when adjustments occur less frequently. This pattern highlights the economic relevance of window selection, as increased turnover may translate into higher transaction costs and reduced net performance. Overall, these findings confirm that the conclusions of the rolling-window analysis are robust to the choice of rebalancing horizon and that the methodology does not rely on a finely tuned window length.

For completeness. Figure 4 represents the Out-of-sample cumulative returns, for different window lengths.

**Figure 4:**
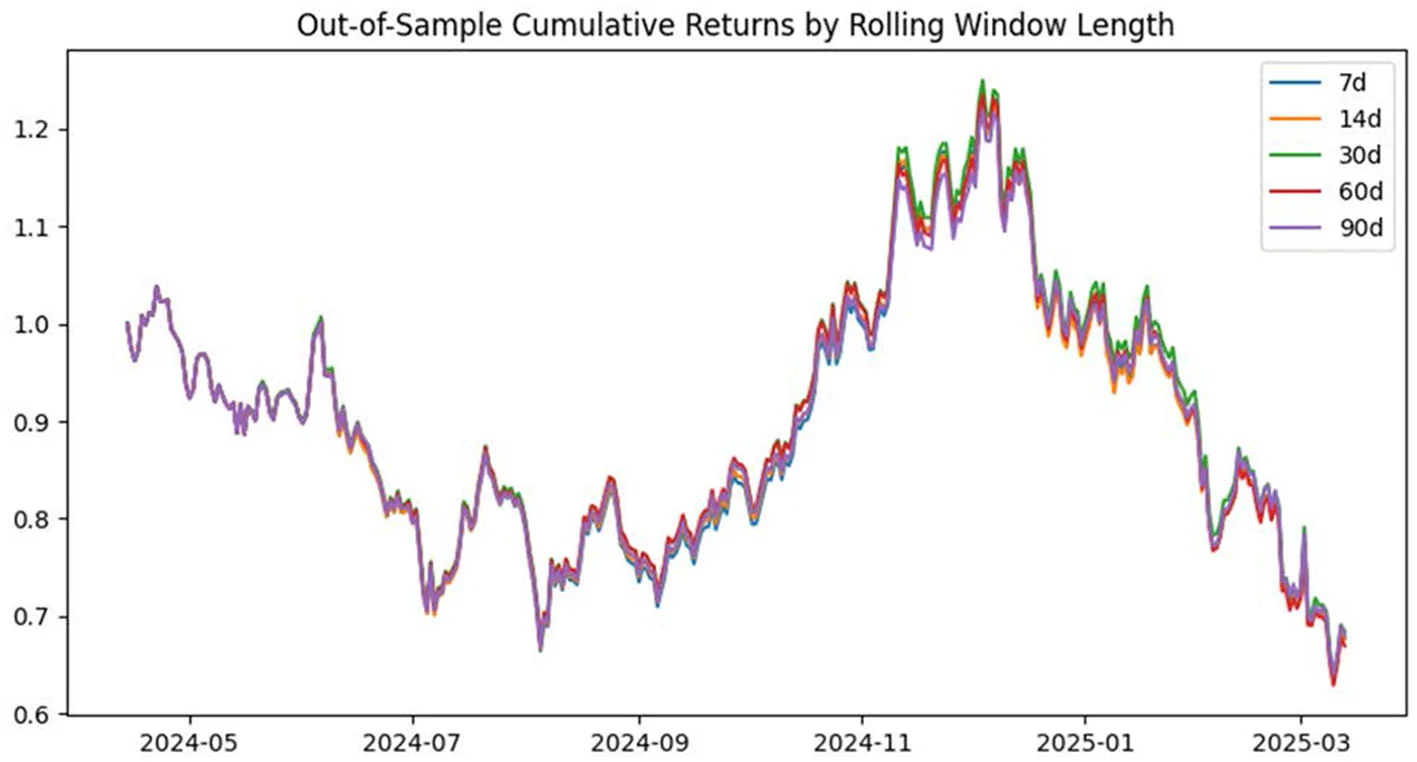
Out-of-sample cumulative returns for different rolling window lengths (7, 14, 30, 60, and 90 days).

Figure 4 visually confirms the robustness of the rolling strategy, showing very similar and almost indistinguishable cumulative return paths across different window lengths.As already highlighted, the most recent period is characterized by negative returns, which visibly decrease the cumulative returns.

## 6 Portfolio evaluation

This section presents the results obtained when an agentic system executes the portfolio strategies described in the previous Section. The evaluation considers both in-sample and out-of-sample performance, using standard financial metrics. The test set corresponds to the latest 20% of observations, 334 rows, while the remaining 80%, 1333 rows, is used as a training set. The main performance metrics analyzed include: Annualized Expected Return, [2] Annualized Volatility, [1] Sharpe Ratio, Sortino Ratio, Maximum Drawdown, Liquidity Risk, Regime Change Detection.

Our aim is to understand which of the two strategies perform better, and how they compare with classical benchmarks. To strengthen the empirical validation of our agentic system, we ask agents to compare the two strategies against five more standard approaches:

- Equal weights portfolio
- Bitcoin (BTC) Buy-and-Hold;
- Ethereum (ETH) Buy-and-Hold;
- Minimum Variance Portfolio;
- Inverse Volatility Portfolio.

The equal weights strategy is the simplest of all. The BTC and ETH portfolios provide passive market benchmarks based on the two largest cryptocurrencies by market capitalization. The Minimum Variance portfolio represents a classical risk-minimization allocation strategy, while the Inverse Volatility portfolio allocates capital proportionally to the inverse of each asset's historical volatility.

All benchmark portfolios are evaluated using the same dataset, train-test split, and performance evaluation metrics employed throughout the MAS framework. This ensures methodological consistency and allows agents to perform a direct comparison between passive investment approaches, traditional portfolio allocation techniques, and the proposed multi-agent strategies.

### 6.1 In-sample performance

We first compare the results achieved during the training phase. Crew A's static optimized portfolio improves over the equal-weighted but remains fixed throughout the investment period. Crew B, with its rolling rebalancing mechanism, significantly enhances the Sharpe ratio while maintaining acceptable volatility. More precisely, Crew A improves the Sharpe ratio from 0.48 (equal-weighted approach) to 0.60 (optimized weights approach). Crew B improves further the results, achieving a Sharpe ratio of 1.00, with reduced volatility (10.2% against 12.4%) and improved expected return (9.9% vs 7.5%).

### 6.2 Out-of-sample performance

To validate the robustness of both strategies, we apply the same logic to the test set. This is a more challenging task, especially considered that the considered test set presents a downward trend of returns, as seen in Figure 4.

Table 6 presents the results of the out-of-sample analysis.

**Table 6:** Comparison of performance metrics between equal-weight portfolios (Report A) and optimized portfolios (Report B) for both crews.

Metric	Equal weights	Crew A	Crew B
Expected return (%)	8	10	10
Volatility (%)	15	12	10
Sharpe ratio	0.53	0.69	0.72
Sortino ratio	0.75	1.10	1.30
Max drawdown (%)	-20	-15	-15
Liquidity risk	–	–	Low
Regime change detection	–	–	No

Table 6 shows that Crew A's Sharpe ratio drops to 0.69, while Crew B maintains a stronger value of 0.72, indicating better out-of-sample generalization. Maximum drawdown is slightly higher for Crew B, reflecting increased exposure to high-return and high-risk assets during certain market phases. Thus, from the evaluation results obtained by executing the two crews independently, Crew B generally outperforms Crew A in the risk-return space, achieving higher risk-adjusted returns while maintaining comparable levels of volatility. This is because the dynamic rebalancing mechanism enables the system to respond to changing market conditions, allocating capital toward outperforming assets and reducing exposure to deteriorating ones.

In addition to the comparison between Crew A and Crew B, in Table 7 we evaluate the proposed MAS-based strategies against the benchmark portfolios introduced at the beginning of this section. This supplementary analysis allows us to assess whether the adaptive optimization performed by the MAS framework generates value beyond simple market exposure and traditional risk-based allocation rules.

**Table 7:** Out-of-sample performance of benchmark portfolios.

Strategy	Expected return (%)	Volatility (%)	Sharpe	Sortino	Max drawdown (%)
BTC buy-and-hold	15.86	42.21	0.38	0.67	-28.30
ETH buy-and-hold	-39.91	56.85	-0.70	-1.14	-60.32
Minimum variance	-0.60	34.22	-0.02	-0.03	-32.71
Inverse volatility	0.00	46.38	0.00	0.00	-47.86

Table 7 shows that benchmark strategies exhibit markedly different risk-return characteristics. BTC Buy-and-Hold achieves the strongest out-of-sample performance among the benchmark portfolios, reflecting the dominant role of Bitcoin within the cryptocurrency ecosystem during the evaluation period. In contrast, ETH Buy-and-Hold delivers negative risk-adjusted performance, while both the Minimum Variance and Inverse Volatility portfolios generate substantially lower returns despite partially reducing portfolio risk.

These findings highlight the difficulty of achieving consistent risk-adjusted performance in cryptocurrency markets, in which returns are highly volatile in time, and provide a broader empirical context for evaluating the effectiveness of MAS-based portfolio construction.

Comparing Table 7 with Table 6, the proposed MAS-based strategies display stronger risk-adjusted performance than the additional benchmark portfolios considered. In particular, Crew B achieves the highest Sharpe and Sortino ratios within the MAS framework, while BTC Buy-and-Hold represents the strongest passive benchmark.

### 6.3 Statistical significance tests

Agentic systems can easily compare alternative portfolio strategies, and related benchmarks, in terms of their returns. They should also assess whether the found return differences among strategies are statistically significant, a step that is often not considered in practical applications.

To assess whether the observed differences in portfolio performance are statistically meaningful, we also perform both parametric and non-parametric tests on the out-of-sample daily returns. Specifically, we apply Welch's two-sample *t*-test Welch (1947), which does not assume equal variances, and the Mann–Whitney U test Mann and Whitney (1947), which is robust to departures from normality. Such tests are commonly employed in empirical finance to assess whether observed performance differences can be attributed to random variation or reflect genuine differences in portfolio behavior Campbell et al. (1997).

Table 8 reports the results obtained when comparing the daily returns generated by Crew A, Crew B, and the equal-weight benchmark portfolio.

**Table 8:** Statistical significance tests on out-of-sample daily returns.

Comparison	Welch t-statistic	Welch p-value	Mann–Whitney U	Mann–Whitney p-value
Crew B vs. Equal weight	-0.200	0.841	46541	0.583
Crew B vs. Crew A	-0.100	0.921	55686	0.971
Crew A vs. Equal weight	-0.127	0.899	46654	0.618

The results reported in Table 8 indicate that none of the pairwise differences in daily portfolio returns are statistically significant at conventional confidence levels. Both the Welch tests and the Mann–Whitney tests yield p-values substantially greater than 0.05.

This evidence suggests that, although the portfolio-level performance metrics reported in Table 6 indicate differences in risk-adjusted performance across strategies, such differences cannot be considered statistically significant when evaluated on daily returns. This result is consistent with the high volatility and noise characterizing cryptocurrency markets and should therefore be interpreted as evidence of limited statistical power rather than evidence of identical portfolio behavior.

To further strengthen the statistical validation of our results, we complement the original Welch and Mann–Whitney tests with paired HAC tests on daily return differences and a time-series bootstrap test on Sharpe ratio differences.

Both procedures confirm that the differences between the Crew strategies and the equal-weight benchmark are not statistically significant at conventional levels. The HAC and bootstrap p-values are above 0.05, and the confidence intervals for the Sharpe ratio differences include zero. Therefore, the observed performance differences should be interpreted with caution (Table 9).

**Table 9:** Robust statistical tests against the equal-weight benchmark.

Comparison	HAC p-value	Sharpe Diff.	Bootstrap p-value	95% CI
Crew A vs EW	0.200	-0.540	0.290	[-1.548, 0.503]
Crew B vs EW	0.204	-0.566	0.295	[-1.606, 0.536]

We remark that statistical significance is not detected at conventional confidence levels, because of the limited sample size and the high volatility of cryptocurrency markets reduce the statistical power of formal hypothesis tests. The observed performance improvements should however be interpreted as indicative, rather than conclusive, evidence of the benefits of adaptive portfolio allocation. Our findings indicate that agentic systems should consider not only to benchmark portfolio strategies against classical benchmarks, but also assess whether the found differences are statistically significant. Often, instead, research studies report that agent-based approaches outperform standards rule-based models across different investment horizons [see Espiga-Fernández et al. (2024)], even in the absence of formal statistical tests.

### 6.4 Ablation and portfolio concentration analysis

To investigate whether the strong portfolio concentration is mainly driven by SOL and SHIB, we perform an additional robustness analysis by excluding these two assets from the investment universe and reapplying both Crew A and Crew B optimization strategies. Table 10 shows the obtained concentration ratios and 12 the resulting performances.

**Table 10:** Portfolio concentration and stability in the ablation analysis.

Strategy	HHI	Effective *N*	Avg. turnover
Crew A – No SOL/SHIB	1.000	1.00	–
Crew B – No SOL/SHIB	0.941	1.08	0.267

The ablation analysis shows that excluding SOL and SHIB does not eliminate concentrated allocations. Crew A converges to a fully concentrated position in ETH, while Crew B reallocates primarily across BTC, ETH and BNB.

An important result emerging from both Crew A and Crew B, also after the robustness check, is the strong concentration of the optimized allocations in a small subset of assets. This outcome is a direct consequence of the Sharpe-ratio maximization objective adopted by the optimization agent. During the considered sample period, these assets exhibited the highest risk-adjusted returns among the available cryptocurrencies, leading the optimizer to allocate most of the portfolio weight to them.

From an economic perspective, concentrated portfolios are not uncommon in unconstrained mean-variance optimization problems originally introduced by Markowitz (1952). As noted by Michaud (1989) and subsequently documented by DeMiguel et al. (2009), such optimization procedures may produce highly concentrated portfolios when expected returns are estimated with error. Small differences in estimated expected returns may translate into large differences in optimal portfolio weights, especially when assets exhibit high correlations, as is the case in cryptocurrency markets. Therefore, the concentration observed in the optimized portfolios reflects both the return characteristics of the selected assets and the limited diversification opportunities available within the considered crypto universe.

At the same time, concentrated allocations may be more sensitive to estimation error and regime changes. For this reason, the results should not be interpreted as a recommendation to allocate exclusively to a few assets in practice. Rather, they highlight the ability of the proposed MAS framework to identify the assets that historically contributed most to portfolio performance under the chosen optimization criterion.

Future research may further improve robustness by introducing diversification constraints, maximum position limits, regularized portfolio optimization methods, or transaction-cost-aware objectives.

## 7 Conclusions

This study has introduced a Multi-Agent System (MAS) for crypto portfolio construction, applied to the top-10 cryptocurrencies using real-world data. By simulating two crew workflows, respectively based on static optimization (Crew A) and rolling window optimization (Crew B), we assessed performance across in-sample and out-of-sample periods.

Crew B's dynamic strategy consistently outperformed the static one, both in terms of return enhancement and risk reduction. Its superior Sharpe and Sortino ratios, coupled with lower volatility and drawdowns, validate the benefits of time-varying allocation when regime shifts occur.

The MAS architecture proved effective in modularizing and automating the entire pipeline: from data preparation and portfolio rebalancing to risk evaluation and documentation. It also ensures transparency, traceability, and replicability, making it suitable for deployment in both academic research and institutional settings.

The results from the evaluation of the portfolios built with agentic AI models confirm the central role of adaptivity in crypto-asset allocation. While Crew A's optimized weights improve the equal-weighted allocation, it remains exposed to structural breaks in volatility [2] and correlations. Crew B's 30-day rolling framework, instead, lifts the in-sample Sharpe ratio [1] from 0.60 to 1.00 and keeps a robust 0.72 out-of-sample, cuts annualized volatility [2] from 12.4% to 10.2%, also maintaining a tolerable volatility out-of-sample of 10%, and limits maximum drawdown to minus 15%. These findings confirm the evidence in Babaei and Giudici (2022), which shows that portfolios updated at high frequency dominate static Markowitz allocations once crypto-market regimes shift.

The results also confirm the importance of including statistical testing of portfolio strategies into agentic systems. Agents should compare portfolio models against classical benchmarks, not only in terms of return statistics, but also in terms of the statistical tests aimed at assessing whether the found differences are significant.

Overall, our study shows that a Multi-Agent System (MAS) architecture provides four tangible advantages:

- **Modularity:** each agent can be upgraded independently, as new data sources or heuristics emerge, exactly as advocated by Babaei and Giudici (2022) for explainable crypto portfolio platforms.
- **Auditability:** the Final Report by the communication agent creates a transparent trail that supervisors can inspect. This directly addresses the black-box criticism of MAS systems. Ensuring transparency, fairness and explainability in ML-based financial systems is not only desirable but necessary, as highlighted by recent guidelines on responsible AI in credit risk modeling Valdrighi et al. (2024).
- **Scalability:** Adding tokens, benchmarks, or transaction costs models requires designing additional agents, rather than rewriting the software code from scratch.
- **Generalisability**: agentic models can easily include comparison with classical benchmarks, including equal weight portfolio, and correlated statistical tests for the difference in returns.

On the other hand, we remark that our study has some limitations, which should be taken into account. In particular:

- **Universe size:** Only the top 10 crypto assets are included.
- **Transaction costs:** Spreads and fees are ignored and could erode part of Crew B's edge.
- **Window length:** A 30-day horizon has been considered. Alternative windows may improve the bias-variance trade-off.

These limitations can be overcome adapting the software code which implements our MAS system, that is available upon request. In particular:

- **Universe size:** Different crypto assets can be considered, and included, without loss of generality;
- **Transaction costs:** Transaction costs can be included, for example as a percentage of trading turnover;
- **Window length:** Different time horizons can be considered, e.g. weekly, bi-monthly or yearly, without loss of generality.

We finally remark that often it may be more useful to predict the direction, and not the price, of financial assets. This could be done in a future work. The work should also be extended to financial market assets, which do not need to take into consideration the specificities of cryptocurrencies, developed in a decentralized financial system. Finally, future work could incorporate stable-coins, regime-switching models, and explainable AI layers, as proposed in the literature [see e.g. Babaei and Giudici (2022) and Giudici et al. (2024)], in order to enhance adaptability and investor trust.

## Data Availability

The data analyzed in this study is subject to the following licenses/restrictions: Available upon request. Requests to access these datasets should be directed to giudici@unipv.it.

## 8 Appendix: CrewAI agents summary

**Table A1:** Summary of CrewAI agents, tools used, and their output artifacts.

Agent name	Tool used	Output
Data Loader Agent	Data Loader Tool	Loads dataset, reports dimensions, saves to a shared file that can be accessed by other agents in the pipeline
Data Cleaner Agent	Data Cleaner Tool	Imputes missing values (NaNs) with the corresponding values from the previous trading day and overwrites the shared csv file with the cleaned dataset
Data Split Agent	Data Splitter Tool	Generates the train and test dataframes in two shared csv files, so each agent will use the same train and test dataset
Portfolio Analyst Agent	Portfolio Metrics Tool	Computes metrics for equal-weight strategy and saves the analysis in a markdown file
Portfolio Optimizer Agent	Portfolio Optimizer Tool	Optimizes weights, saves to optimized_weights.csv
Portfolio Metrics Benchmark Agent	Portfolio Metrics with Benchmark Tool	Evaluates portfolio with benchmark and saves the analysis into two markdown files (optimized_train_metrics.md, optimized_test_metrics.md)
Final Report Agent	No tools	Integrates prior reports into a complete and final report (final_portfolio_analysis_report.md)
